# Ultrafast Charge Carrier Dynamics in InP/ZnSe/ZnS Core/Shell/Shell Quantum Dots

**DOI:** 10.3390/nano12213817

**Published:** 2022-10-28

**Authors:** Shijia Zeng, Zhenbo Li, Wenjiang Tan, Jinhai Si, Yuren Li, Xun Hou

**Affiliations:** Key Laboratory for Physical Electronics and Devices of the Ministry of Education, Shaanxi Key Laboratory of Information Photonic Technique, School of Electronics Science and Engineering, Xi’an Jiaotong University, 28 Xianning Road, Xi’an 710049, China

**Keywords:** InP/ZnSe/ZnS quantum dots, ultrafast carrier dynamics, femtosecond transient absorption, light-emitting diodes

## Abstract

The excellent performance of InP/ZnSe/ZnS core/shell/shell quantum dots (CSS-QDs) in light-emitting diodes benefits from the introduction of a ZnSe midshell. Understanding the changes of ultrafast carrier dynamics caused by the ZnSe midshell is important for their optoelectronic applications. Herein, we have compared the ultrafast carrier dynamics in CSS-QDs and InP/ZnS core/shell QDs (CS-QDs) using femtosecond transient absorption spectroscopy. The results show that the ZnSe midshell intensifies the electron delocalization and prolongs the in-band relaxation time of electrons from 238 fs to 350 fs, and that of holes from hundreds of femtoseconds to 1.6 ps. We also found that the trapping time caused by deep defects increased from 25.6 ps to 76 ps, and there were significantly reduced defect emissions in CSS-QDs. Moreover, the ZnSe midshell leads to a significantly increased density of higher-energy hole states above the valence band-edge, which may reduce the probability of Auger recombination caused by the positive trion. This work enhances our understanding of the excellent performance of the CSS-QDs applied to light-emitting diodes, and is likely to be helpful for the further optimization and design of optoelectronic devices based on the CSS-QDs.

## 1. Introduction

Colloidal quantum dots (QDs) have attracted great attention in the field of light-emitting diodes (LEDs) [1,2,3,4,5,6,7,8,9] due to their tunable band gap in the visible light range, excellent emission performance, flexible processability and low cost [10,11,12]. However, most of the studied materials are Cd-based QDs, which can cause severe environmental issues and diseases due to their high toxicity [13,14,15]. InP-based QDs, which have recently made a breakthrough in LEDs with the maximum external quantum efficiency of 21.4% [16], are expected to become the best low-toxicity alternative to Cd-based QDs [5].

The excellent emission characteristics of InP-based QDs benefit from the appropriate passivation shell growth on the InP surface, which can not only improve the luminescence efficiency, but also enhance their stability. ZnS is a commonly used shell material due to its good chemical stability and larger band gap than InP, which leads to a good quantum confinement effect [17,18,19]. However, the large lattice mismatch of 7% between ZnS and InP will lead to more interface defects, which is not conducive to luminescence. Further, the introduction of ZnSe midshell can effectively improve the interface defects, due to the small lattice mismatch of 3.3% between ZnSe and InP [5]. The nearly 100% emission efficiency of InP/ZnSe/ZnS core/shell/shell QDs is an important premise for InP/ZnSe/ZnS QD-based LEDs to achieve excellent performance [20]. However, compared to Cd-based QDs, their luminescence properties still need to be improved to obtain better device efficiency and long-term stability [21]. The research on the photophysical process of the CSS-QDs is expected to provide insights into the improvement of the device performance.

Recent studies have shown that the ZnSe midshell in the CSS-QDs can effectively suppress the negative trion Auger recombination process, which acts as one of the limiting factors on LED performance [22,23,24,25,26]. Moreover, the research shows that the ZnSe phonons are closely related to the energy gap between the band-edge and defect states, and even participate in the carrier capture process in CSS-QDs [21]. In addition, it is found that the hot carrier dynamics in the CSS-QDs are affected by the ZnSe midshell morphology [27]. However, there is still a lack of basic and comprehensive studies of ultrafast carrier dynamics in CSS-QDs by femtosecond transient absorption (TA) technology, which is usually used to understand the operating mechanism of photoelectric materials in devices [28,29]. Moreover, a comparative dynamics study between CS-QDs and CSS-QDs is essential to understanding the luminescence benefits of CSS-QDs in LEDs caused by the ZnSe midshell.

Here, the ultrafast carrier dynamics of InP-based CS-QDs and CSS-QDs are studied comparatively by femtosecond TA spectroscopy and time-resolved photoluminescence (TRPL) techniques. The results show that there are lower carrier relaxation times and trapping times in the CSS-QDs compared with the CS-QDs due to the enhanced exciton delocalization in CSS-QDs. It was also observed that the density of higher-energy hole states in the valence band of the CSS-QDs increases significantly, which may lead to their obvious advantage in preventing positive trion Auger recombination events. In addition, the PL dynamics process indicates that the defect emission in CSS-QDs was obviously weakened due to a lower lattice mismatch. These results are helpful in understanding the excellent photoelectric characteristics of CSS-QDs.

## 2. Materials and Methods

### 2.1. Materials

The InP/ZnS QDs and InP/ZnSe/ZnS QDs were obtained from Mesolight Inc. (Suzhou, China). Anthraquinone (98%, Macklin) and Toluene (99.8%, Sinopharm Chemical Reagent Co., Ltd., Singapore) were used as received.

### 2.2. Characterization

The transmission electron microscope (TEM) images were obtained using Talos F200X with the voltage of 200 kV. X-ray diffraction (XRD) patterns were recorded by a Bruker D8 Advance. Ultraviolet–visible (UV-vis) absorption spectra were recorded by a UV-2600 and the photoluminescence (PL) spectra were taken with an FLS920 (Edinburgh) spectrophotometer. The TRPL spectra and photoluminescence quantum yields (PLQYs) were recorded at 450 nm excitation by the FLS980 (Edinburgh) equipment, which integrates a time-correlated single-photon counting (TCSPC) system and a switchable integrating sphere lined with polytetrafluoroethylene.

### 2.3. Femtosecond TA Spectroscopy

A femtosecond laser beam (Vitesse, Conherent, 1 kHz repetition rate, 100 fs pulse width) with the central wavelength of 800 nm was first split into two beams. One of them was converted into 400 nm by a BBO frequency-doubling crystal, and then modulated to 500 Hz with an optical chopper as the pump light. The other beam was focused into a sapphire crystal to generate a supercontinuum white light as the probe light. Then, the pump and the probe beams were focused and overlapped well in the sample. Since the repetition rates of the probe light and pump light were 1 kHz and 500 Hz, respectively, the TA spectra were obtained by comparing the probe light spectra with and without pump light excitation.

## 3. Results

**Figure 1:**
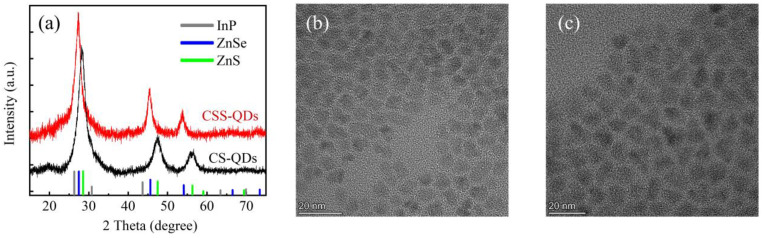
(**a**) XRD patterns for the CS-QDs and CSS-QDs. The vertical lines below indicate the diffraction peak positions of zinc-blended InP (gray), ZnS (green) and ZnSe (blue). (**b**,**c**) TEM images of both CS-QDs and CSS-QDs, respectively.

The XRD patterns (Figure 1a) have been measured to verify the structural difference between the CS-QDs and CSS-QDs. The diffraction peaks of the CS-QDs correspond well with those of zinc-blended ZnS (JCPDS No. PDF 77-2100), indicating that the ZnS shell is coated on the InP core [30]. It was also observed that the diffraction peaks of the CSS-QDs correspond well with those of zinc-blended ZnSe (JCPDS No. PDF 80-0021), indicating the existence of a ZnSe midshell [31]. Since the same InP core was used to prepare the two materials, the existence of a ZnSe midshell results in a larger size of CSS-QDs compared with CS-QDs, as shown in Figure 1b,c. The particle sizes of CS-QDs and CSS-QDs are 6.3 ± 0.6 nm and 8.7 ± 0.7 nm, respectively (see Figure S1 for the size distribution), which suggests that the thickness of the ZnSe midshell is about 2.4 nm.

**Figure 2:**
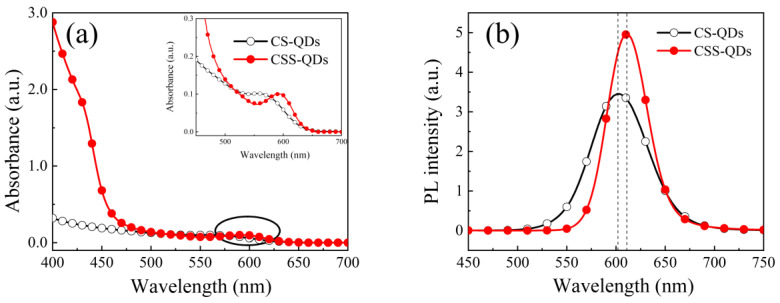
(**a**) UV-vis absorption spectra of both CS−QDs and CSS−QDs. The inset shows the details of band-edge absorption peaks of CS−QDs and CSS−QDs. (**b**) PL spectra of both CS−QDs and CSS−QDs.

The UV-vis absorption spectra of the CS-QDs and CSS-QDs are displayed in Figure 2a. It was found that the band-edge absorption peaks of the CS-QDs and CSS-QDs are about 573 nm and 595 nm, respectively. The obvious red shift of the absorption peak in the CSS-QDs is attributed to the enhanced electron delocalization from the InP core to the ZnSe midshell [22,32]. Moreover, a continuously enhanced absorption of the CSS-QDs from 450 nm to a shorter wavelength was also observed, and the absorption peak at around 430 nm is close to the absorption of the ZnSe [33], which indicates that the enhanced absorption in CSS-QDs can be attributed to the ZnSe midshell. In addition, the lattice mismatch between InP and ZnS can be compromised by the ZnSe midshell, thus resulting in a stronger emission for the CSS-QDs, as shown in Figure 2b. The PLQY values of the CSS-QDs and the CS-QDs are 87% and 63%, respectively, indicating that the core/shell/shell structure has improved the defect passivation. The emission peak of the CSS-QDs shows significant red shift compared with that of the CS-QDs, which can be attributed to the larger exciton delocalization in CSS-QDs caused by the ZnSe midshell.

**Figure 3:**
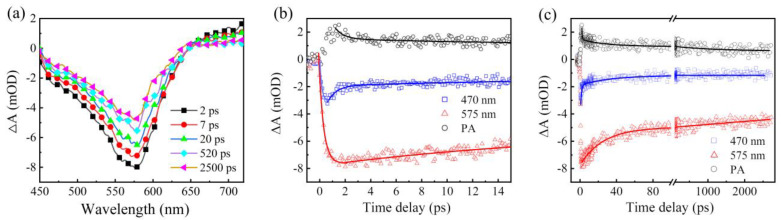
(**a**) TA spectra of CS−QDs pumped at 400 nm. The formation process (**b**) and decay dynamics (**c**) at 575 nm, 470 nm and PA extracted from (**a**). The solid lines are fit curves.

The ultrafast carrier dynamics between the CS-QDs and CSS-QDs were further compared to confirm the influence of the ZnSe midshell on the emission process. Figure 3a shows the femtosecond TA spectra of the CS-QDs pumped at 400 nm. The pump fluence of a single pulse was controlled at about 70 $\mu J/{\mathrm{cm}}^{2}$ to avoid the multi-exciton process [33]. The signal near 575 nm is consistent with the wavelength of the first exciton absorption peak in the absorption spectrum, which can be considered as a band-edge bleaching signal. In addition, there is a weaker bleaching signal near 470 nm that exists for more than 3 ns. Similar short wavelength signals have been widely reported [33,34,35,36], which are generally considered to be composed of higher-energy hole states and band-edge electron states. The density distribution of the valence band states allows the occupancy probability of holes to be distributed on these higher-energy states, meaning that these holes on the high-energy states do not rapidly relax to the band-edge states [22]. Moreover, a broad-band photoinduced absorption (PA) signal above 650 nm was observed, which is usually described as the transition of the band-edge state to higher levels or defect states [34,37]. The formation and decay dynamics of 470 nm, 575 nm and PA signals were then extracted into Figure 3b,c. According to the fitting of the single-exponential function, the formation times at 470 nm and 575 nm are 238 ± 5 fs and 357 ± 3 fs, respectively. The bleaching signal is related to the state filling of electrons and holes, and the formation time is generally considered as the time at which the excited electrons and holes relax to their corresponding states [33,35]. After the QDs are excited, the excited electrons relax to the band-edge state, and the excited holes relax to the higher-energy state to form the 470 nm signal, as described above (also refer to orange curve arrow inenergy level diagram below. Because the time required for the relaxation of the excited holes to the higher-energy state is very short [33], the time constant of 238 fs for the 470 nm signal can be attributed to the time of the excited electrons relaxing to the band-edge state. The formation time for the 575 nm signal is related to the time of the excited electrons and holes relaxing to the band-edge state. This time is slightly longer than the formation time for the 470 nm signal, which may be due to the longer time required for the excited holes to relax to the band-edge state, while the PA signal and 575 nm signal reached the peak at almost the same time, which once again shows that the PA signal was related to the band-edge state.

Subsequently, the decay dynamics of these signals were fitted, and it was found that the signal of 575 nm had two decay components with the time constants of 25.6 ± 1.4 ps ($\tau_{1-575\;\mathrm{nm}}^{\mathrm{cs}}$) and >3 ns ($\tau_{2-575\;\mathrm{nm}}^{\mathrm{cs}}$), respectively. The signal of 470 nm has three decay components with time constants of 1± 0.1 ps ($\tau_{1-470\;\mathrm{nm}}^{\mathrm{cs}}$), 29.5 ± 3.1 ps ($\tau_{2-470\;\mathrm{nm}}^{\mathrm{cs}}$) and >3 ns ($\tau_{3-470\;\mathrm{nm}}^{\mathrm{cs}}$), respectively. Since the exciton lifetime in InP QDs is about 30 ns [16,20,22], the decay components of the bleach signals with a time constant less than 1 ns were usually attributed to the defects trap or power-dependent Auger process. It has been proven that there is no multi-exciton generated in CS-QDs when the pump fluency is close to 70 $\mu J/{\mathrm{cm}}^{2}\;$ [33], and further power-dependent TA spectra were found, suggesting that the decay process of the 575 nm signal had hardly changed (Figure S2), so the $\tau_{1-575\;\mathrm{nm}}^{\mathrm{cs}}$ was attributed to the time of the electron trap [38,39]. The $\tau_{2-575\;\mathrm{nm}}^{\mathrm{cs}}$ was vaguely considered as the time of exciton recombination or long-term defect trap, due to the lack of a complete detection process. The $\tau_{2-470\;\mathrm{nm}}^{\mathrm{cs}}$ is very close to the $\tau_{1-575\;\mathrm{nm}}^{\mathrm{cs}}$, and considering that both the 470 nm signal and the 575 nm signal share the band-edge electronic states, $\tau_{2-470\;\mathrm{nm}}^{\mathrm{cs}}$ is also assigned as the time of the electron trap. The $\tau_{1-470\;\mathrm{nm}}^{\mathrm{cs}}$ may be the trap time of higher-energy hole states. Previous reports have shown that there are some hole defects in the InP QDs above the valence band-edge, and some holes will be trapped during cooling relaxation [33,40]. The $\tau_{3-470\;\mathrm{nm}}$ was considered to have the same time constant as $\tau_{2-575\;\mathrm{nm}}^{\mathrm{cs}}$. PA signals were also fitted with three decay components with the time constants of 0.5 ± 0.2 ps, 23.6 ± 5 ps and >3 ns, respectively. Among them, the fastest decay component may be related to the trapping of band-edge holes [34,37], and the second decay component was also assigned to the electron trap process, while the slowest decay component was considered to be the same process as in $\tau_{2-575\;\mathrm{nm}}^{\mathrm{cs}}$.

**Figure 4:**
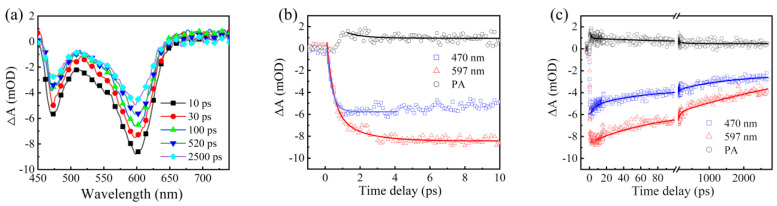
(**a**) TA spectra of the CSS−QDs pumped at 400 nm. The formation process (**b**) and decay dynamics (**c**) at 597 nm, 470 nm and PA extracted from (**a**). The solid lines are fit curves.

Figure 4a shows the temporal evolution of the femtosecond TA spectra of CSS-QDs after photoexcitation at 400 nm, where signals near 597 nm were considered as the band-edge bleaching signal due to the wavelength corresponding to the first exciton absorption peak in Figure 2a. Meanwhile, an obvious bleaching signal was observed near 470 nm. According to the above discussions of the CS-QDs, it was also considered here that the 470 nm signal in the CSS-QDs is formed by the higher-energy holes and band-edge electrons states. The PA signals above 680 nm were considered to have a similar origin to the PA signal in CS-QDs. The formation and decay dynamics of 470 nm, 597 nm and PA signals were then extracted into Figure 4b,c. According to the fitting of exponential function, the formation time of 470 nm is 350 ± 3 fs, while the formation process of 597 nm consists of two components, whose time constants are 380 ± 7 fs and 1.6 ± 0.6 ps, respectively. The time constant of 380 fs is close to the formation time of 470 nm, which is considered to be the relaxation time of electrons. The slower 1.6 ps is thus related to the hole relaxation time [22,33]. This seems to be somewhat different from the formation process of CS-QDs, including the subsequent decay dynamics process, which will be further described later.

By fitting the decay dynamics process of these signals in CSS-QDs, the decay time constants of the 597 nm signal are 76 ± 5 ps ($\tau_{1-597\;\mathrm{nm}}^{\mathrm{css}}$) and >3 ns ($\tau_{2-597\;\mathrm{nm}}^{\mathrm{css}}$), respectively, and those of the 470 nm signal are 5.5 ± 2.1 ps ($\tau_{1-470\;\mathrm{nm}}^{\mathrm{css}}$), 66 ± 8 ps ($\tau_{2-470\;\mathrm{nm}}^{\mathrm{css}}$) and >3 ns ($\tau_{3-470\;\mathrm{nm}}^{\mathrm{css}}$), respectively. According to the previous report [22], the average number of excitons generated in CSS-QD ($\langle N_{0}\rangle$) can be estimated using the relationship $\langle N_{0}\rangle =\sigma_{\mathrm{abs}}j,\;$ where $\sigma_{\mathrm{abs}}$ is the absorption cross-section and j is the pump fluence. The $\langle N_{0}\rangle$ for the CSS-QDs was estimated to be about 0.28 under the pump fluence of 70 $\mu J/{\mathrm{cm}}^{2}$, which is far less than 1. In addition, we also performed a TA measurement at the other two pump fluences, and the theoretical calculated values of the $\langle N_{0}\rangle$ under these two conditions were also less than 1. The experimental results show that the dynamic curves of the 597 nm signal at these pump fluences are almost the same (Figure S3), which indicates that there is no Auger process in the CSS-QDs in our experiment. Therefore, the $\tau_{1-597\;\mathrm{nm}}^{\mathrm{css}}$ and the $\tau_{2-470\;\mathrm{nm}}^{\mathrm{css}}$ were attributed to the electron trapping time here. The $\tau_{1-470\;\mathrm{nm}}^{\mathrm{css}}$ was considered as the trap time of higher-energy hole states, and the $\tau_{2-597\;\mathrm{nm}}^{\mathrm{css}}$ and $\tau_{3-470\;\mathrm{nm}}^{\mathrm{css}}$ were considered as the time of exciton recombination or long-term defect trap, according to the above description.

## 4. Discussion

When comparing the TA spectra of the CS-QDs and the CSS-QDs, the significant difference is that the bleach signal near 470 nm significantly enhances the CSS-QDs. This is caused by the existence of the ZnSe midshell. Considering that the bulk band gap of ZnSe is 464 nm, and its valence band states correspond to higher-energy hole states in the InP core [33], the introduction of the ZnSe midshell may increase the density of higher-energy hole states in CSS-QDs. Naturally, what effect does the enhanced bleach signal at 470 nm have on the fluorescence emission? Since no PL peak was observed near 470 nm, the radiative recombination between band-edge electrons and higher-energy holes can be ruled out. On the contrary, if the non-radiative recombination event occurs between the band-edge electrons and the higher-energy holes, the PL performance of CSS-QDs should be seriously reduced, which is inconsistent with the higher PLQY. Therefore, a possible mechanism is that the higher-energy hole states are equivalent to the storage energy level of hole, that is, the photogenerated holes will not all relax to the valence band-edge within tens of picoseconds, which is usually the time scale of Auger recombination [11,41]. The existence of higher-energy hole states can preclude the emergence of excessive hole population density at the valence band-edge, thus reducing the probability of Auger recombination with positive trion. This explanation is supported by recent reports, which state that the Auger recombination with positive trion can be effectively suppressed by the midshell [16,42]. The pump fluence-dependent TA spectra were further assessed for the CSS-QDs, as shown in Figure S4. The signal strength of 470 nm will become stronger with the increasing pump power, and the increasing amplitude of the signal at 470 nm even exceeds that of the signal at 597 nm. This indicates that with the increasing number of photogenerated holes, relatively more holes will reach higher-energy levels, resulting in greater signal enhancement at 470 nm. This shows the ability of higher-energy hole states as hole storage energy levels to prevent positive trion Auger recombination events, which increases the advantages of the CSS-QDs in the application of QD-based LEDs.

**Figure 5:**
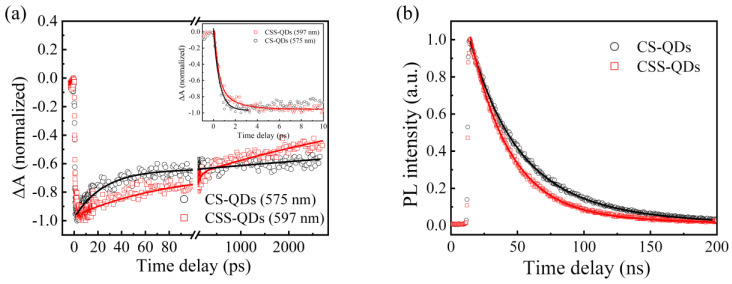
Normalized dynamics of band-edge bleach signals (**a**) and PL (**b**) for the CS−QDs and CSS−QDs.

It can be noted that there are also great differences in the band-edge dynamics between the CS-QDs and the CSS-QDs. The normalized band-edge dynamics curves are shown in Figure 5a. The formation process of the CSS-QDs is obviously slower than that of CS-QDs, which is caused by the partial electron delocalization from the InP core to ZnSe midshell. The small conduction band offset at the InP and ZnSe interface means that the electron wave function lies within the ZnSe midshell [33,43,44], but the hole is still confined at InP core [16]. This will affect the relaxation process of photogenerated excitons. On the one hand, according to Fermi’s golden rule, the in-band relaxation time of electrons in CSS-QDs will be slower due to the decreased wave function overlapping with electron states in InP core. On the other hand, the hole relaxation in the InP core is also hindered by the small wave function overlap, although phonon scattering is the main mechanism for the hole relaxation. This prolongs hole relaxation time from hundreds of femtoseconds to about 1.6 ps, which is consistent with recent reports [22,33].

In addition to the difference in the formation process of the band-edge bleaching signal between the CS-QDs and the CSS-QDs, the difference in their recovery dynamics is also noteworthy. The defect trapping time constant (76 ps) of the CSS-QDs is significantly longer than that of CS-QDs (25.6 ps). On the one hand, electron delocalization leads to an increase distance between electrons and the defect state. On the other hand, the introduction of the midshell will lead to an increase in the energy difference between the band-edge states and defect states [21]. According to the energy gap law, this will lead to a reduction in the trapping rate. Moreover, the contributions of electron trapping components to recovery dynamics in the CSS-QDs and CS-QDs are 26% and 31%, respectively, and this indicates that CSS-QDS has relatively few defect states. However, this does not seem to be consistent with the fact that CSS-QDs has a significantly higher PLQY (87%) than CS-QDs (63%). According to previous reports, although the shell can play a role in passivating InP QDs, some inherent surface defects or the local oxidation of InP QDs will still remain in the new core/shell structure as deep defects [16,45,46,47]. Therefore, the defect states observed in the TA spectra are likely to be inherent deep defects of QDs, and the passivation of the ZnSe midshell for these deep defects is very limited. To further understand these mechanisms, a TRPL test was performed, as shown in Figure 5b. The two curves are reproduced using the bi-exponential function, wherein the decay time constants (and its contribution) of the CS-QDs are 32 ns (70%) and 78 ns (30%), and those of the CSS-QDs are 30 ns (95%) and 77 ns (5%). It can be seen that the decay time constants of the CS-QDs and the CSS-QDs are very close, in which the faster components of 30 ns are considered as single exciton emission, and the slower components of more than 70 ns are considered defect emissions, which are considered as shallow defects related to lattice mismatch [5,16,22]. The lower amount of defect emissions in CSS-QDs indicates that these shallow defects were effectively passivated by the ZnSe midshell. Therefore, by comparing the dynamics process of the band-edge bleach signal and TRPL, it can be found that the ZnSe midshell can effectively passivate the shallow defects related to lattice mismatch, but it is difficult to effectively passivate the inherent deep defects in InP QDs. This shows that the initial quality of the InP core in the CSS-QDs is also very important.

**Figure 6:**
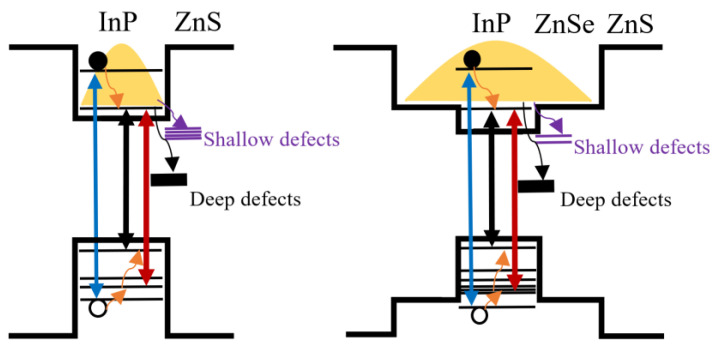
Schematic illustration of the relaxation process of photogenerated carriers in CS−QDs and CSS−QDs. For both, the blue two-way arrow indicates photoexcitation at 400 nm. The black and red two-way arrows represent the band-edge signals and short wavelength signals in the TA spectrum. The orange wavy arrow represents the in-band relaxation process of hot carriers, the purple wavy arrow represents the transition of electrons to shallow defects and the black wavy arrows indicate the transition of electrons to deep defects.

Based on the above analysis, we propose a possible energy level structure and photogenerated exciton dynamics model for the CSS-QDs and the CS-QDs in Figure 6. The red two-way arrows indicate the band-edge signal, and the black two-way arrows represent the signal near 470 nm. The main difference between the two materials is that as the degree of electron delocalization is enhanced, the density of higher-energy hole states increases and that of defect states decreases in the CSS-QDs. When the CSS-QDs was excited by a 400 nm pulse laser, photogenerated electrons and holes were excited to a higher energy level. Subsequently, electrons relax to the conduction band-edge within about 380 fs, and holes first rapidly relax to the higher-energy hole states, and then holes partially relax to the valence band-edge within about 1.6 ps. When the electrons relaxed to the band-edge, some of them were trapped by deep defects within 76 ps. Some electrons were trapped by the shallow defects, and then recombined with holes within 70 ns. The remaining band-edge electrons were radiatively recombined with the band-edge holes within 30 ns.

## 5. Conclusions

The ultrafast carrier dynamic process of InP/ZnSe/ZnS QDs and InP/ZnS QDs are here comparatively studied. The results show that the introduction of the ZnSe midshell brings the following advantages. First, the trapping time constants of the deep defect were significantly increased from 25.6 ps to 76 ps, due to the increased degree of electron delocalization from the InP core to the ZnSe midshell. Moreover, the density of shallow defect states was significantly reduced due to the small lattice mismatch of 3.3% between the InP core and the ZnSe shell. Finally, the ZnSe midshell increased the density of higher-energy hole states, which can prevent the excessive concentration of the hole population at the valence band-edge, and thus is beneficial to inhibiting the Auger recombination caused by the positive trion. These results are expected to be helpful for understanding the excellent performance of InP/ZnSe/ZnS QDs in LEDs, and also provide valuable insights into the design and application of optoelectronic devices based on InP/ZnSe/ZnS QDs.

## Data Availability

The data presented in this study are available on request from the corresponding author.

## Supplementary Materials

The following supporting information can be downloaded at https://www.mdpi.com/article/10.3390/nano12213817/s1—Figure S1: the size distributions of CS-QDs and CSS-QDs; Figure S2: Power-dependent TA spectra of decay dynamics for the CS-QDs at 575 nm. Figure S3: Power-dependent TA spectra of decay dynamics for the CSS-QDs at 597 nm. Figure S4: 400 nm excited TA spectra of CSS-QDs at different pump-fluences normalized at band-edge bleach maximum.
