# Supplementary material for: Ultrafast Charge Carrier Dynamics in InP/ZnSe/ZnS Core/Shell/Shell Quantum Dots
Source: Nanomaterials (Basel). 2022 Oct 28;12(21):3817. doi: 10.3390/nano12213817 (PMC9657385; doi:10.3390/nano12213817)
Supplement: Supplementary file 1 [file nanomaterials-12-03817-s001.zip › nanomaterials-1985498-supplementary.pdf]

nanomaterials

Supplementary Materials

Ultrafast Charge Carrier Dynamics in InP/ZnSe/ZnS Core/Shell/Shell Quantum Dots

Shijia Zeng, Zhenbo Li, Wenjiang Tan *, Jinhai Si, Yuren Li and Xun Hou

Key Laboratory for Physical Electronics and Devices of the Ministry of Education, Shaanxi Key Laboratory of Information Photonic Technique, School of Electronics Science and Engineering, Xi'an Jiaotong University, 28 Xianning Road, Xi'an 710049, China

* Correspondence: tanwenjiang@mail.xjtu.edu.cn

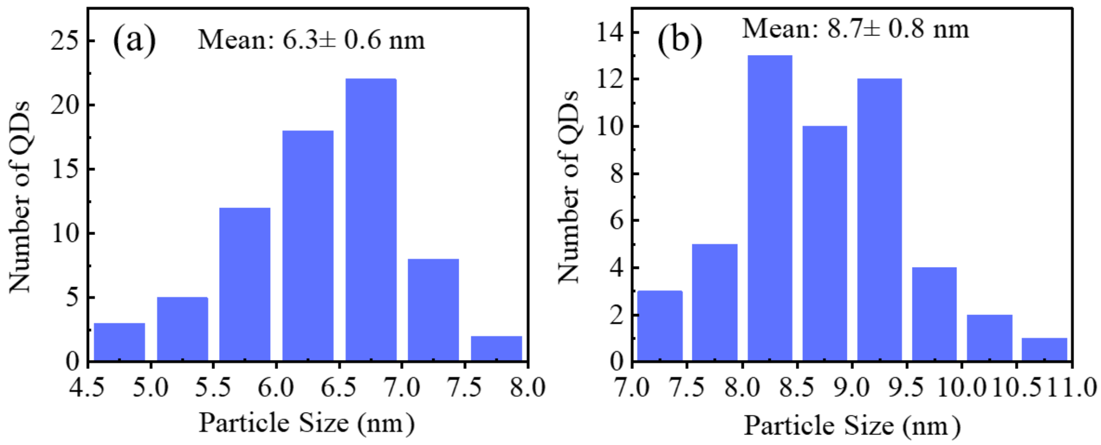

Figure S1. (**a**) and (**b**) are the size distributions of CS-QDs and CSS-QDs corresponding to Figure 1b and 1c.

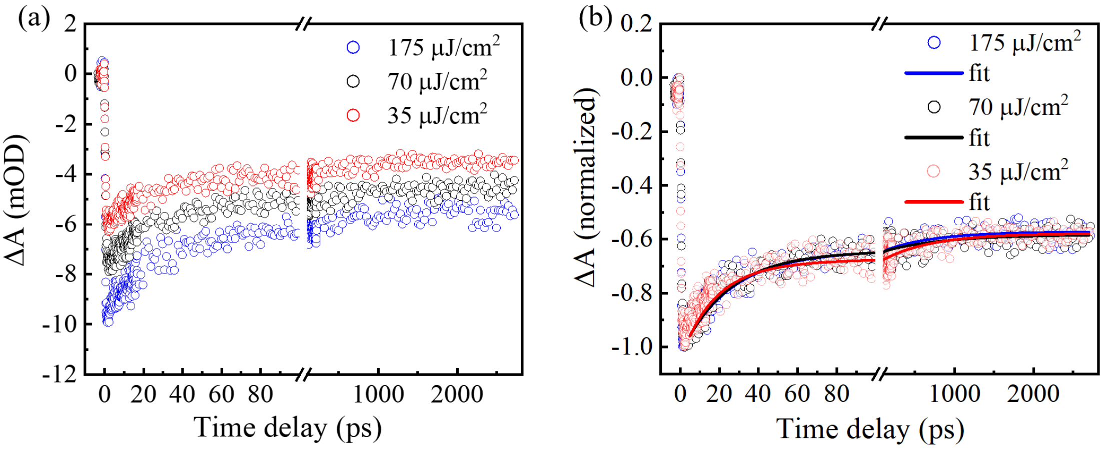

Figure S2. (**a**) Power dependent TA spectra of decay dynamics for the CS-QDs at 575 nm. (**b**) Normalized decay dynamics of (**a**).

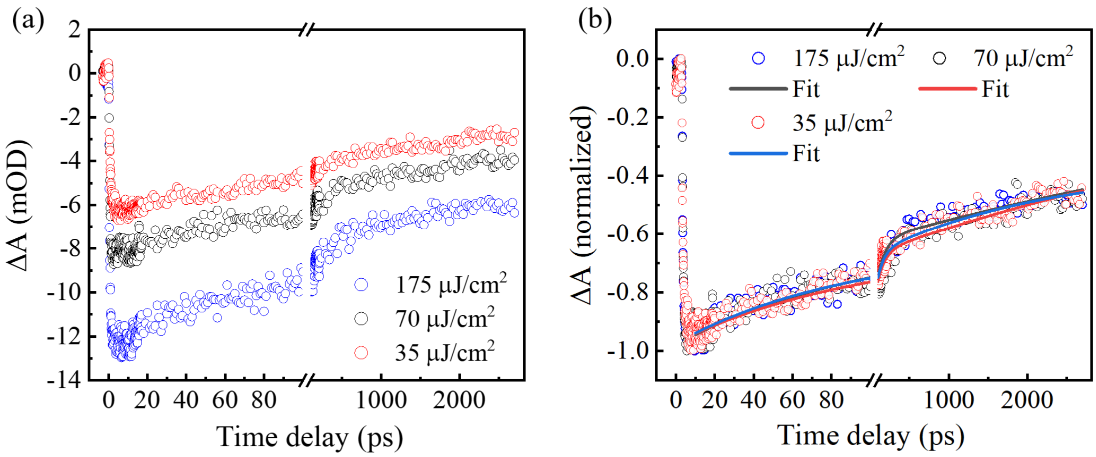

Figure S3. (**a**) Power dependent TA spectra of decay dynamics for the CSS-QDs at 597 nm. (**b**) Normalized decay dynamics of (**a**).

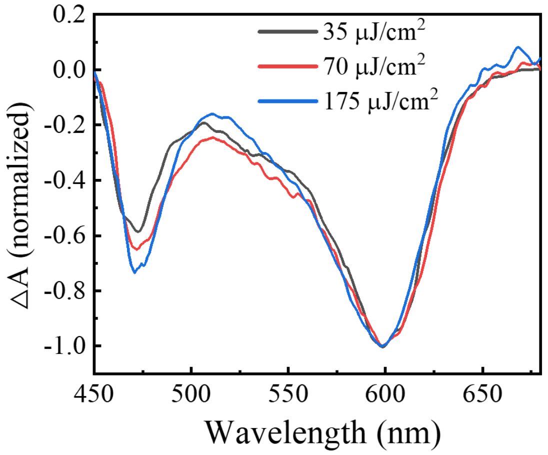

Figure S4. 400 nm excited TA spectra of CSS-QDs at difference pump-fluence normalized at band-edge bleach maximum.
